# Serum Levels of Growth Differentiation Factor 11 Are Independently Associated with Low Hemoglobin Values in Hemodialysis Patients

**DOI:** 10.1089/biores.2016.0015

**Published:** 2016-06-01

**Authors:** Sho-ichi Yamagishi, Takanori Matsui, Yuka Kurokawa, Kei Fukami

**Affiliations:** ^1^ Department of Pathophysiology and Therapeutics of Diabetic Vascular Complications, Kurume University School of Medicine, Kurume, Japan.; ^2^ Division of Nephrology, Department of Medicine, Kurume University School of Medicine, Kurume, Japan.

**Keywords:** aging, cardiology, regeneration

## Abstract

Circulating levels of growth differentiation factor 11 (GDF11) have been shown to decrease with age in several mammalian species, and supplementation of GDF11 by heterochronic parabiosis or systemic administration reverses age-related organ damage. However, there is some controversy about the pathophysiological role of GDF11 in aging-associated organ damage. Since aging process is accelerated in uremia, we compared serum levels of GDF11 in hemodialysis (HD) patients with those in age-matched healthy controls, and then determined the independent clinical correlates of GDF11 in HD subjects. Sixty-two maintenance HD patients (34 male and 28 female; mean age, 52.6 years; mean duration of HD, 7.7 months) were enrolled in the present study. Twenty-nine age-matched subjects were used as a control. GDF11 was measured by a commercially available enzyme-linked immunosorbent assay kit. Serum GDF11 levels in HD patients were significantly higher than those in controls (9.4 ± 5.1 pg/mL vs. 7.3 ± 5.9 pg/mL). A statistical significance was demonstrated between GDF11 and hemoglobin (inversely). Multiple stepwise regression analysis revealed that hemoglobin (*p* < 0.001) was a sole independent correlate of GDF11 levels in HD patients (*R*^2^ = 0.168). Our present study suggests that kinetics and regulation of circulating GDF11 may differ between normal physiological aging process and accelerated pathological aging conditions, such as uremia. Given that GDF11 has been shown to inhibit erythroid maturation in mice, elevation of GDF11 levels may be involved in erythropoietin-resistant anemia in HD patients.

## Introduction

Growth differentiation factor 11 (GDF11), one of the members of bone morphogenetic protein/transforming growth factor-β superfamily, is first identified as a regulator of axial skeletal patterning during early embryogenesis, and then found to play a crucial role in various cellular processes, including kidney organogenesis and tissue homeostasis.^1–3^ Recently, systemic levels of GDF11 have been shown to decrease with age in several mammalian species, and supplementation of GDF11 reverses age-related cardiac hypertrophy, structural and functional derangements of skeletal muscle, and dysfunction of neurogenesis.^4–7^ Furthermore, higher levels of GDF11 and/or its homologue GDF8 are associated with lower risk of cardiovascular events and total mortality in patients with stable ischemic heart disease (IHD).^8^ These observations suggest that GDF11 may be a circulating rejuvenation factor that restores youthful characteristics in various aged organs, thereby being a novel therapeutic target for antiaging medicine. However, there is some controversy about the pathophysiological role of GDF11 in aging-associated organ damage.^9–11^ Indeed, Smith et al. reported that GDF11 did not rescue cardiac hypertrophy in aged mice.^9^ Moreover, GDF11 has been shown to increase *rather than* decrease with age in both rats and humans, and to inhibit toxin-damaged muscle regeneration in old mice.^10,11^

Aging process is accelerated in uremia, and aging-related organ derangements, including sarcopenia, cardiac hypertrophy, and impaired cognitive function, are more prevalent in uremic patients.^12,13^ Therefore, to clarify the pathophysiological role of GDF11 in humans, it is interesting to examine whether circulating levels of GDF11 are decreased or increased in uremic subjects. In this study, we compared serum levels of GDF11 in hemodialysis (HD) patients with those in age-matched healthy controls, and then determined the independent clinical correlates of GDF11 in subjects on HD.

## Subjects and Methods

Sixty-two maintenance HD patients (34 male and 28 female; mean age, 52.6 years; mean duration of HD, 7.7 months) were enrolled in this study. Patients were dialyzed for 4–5 h with high-flux dialyzers three times a week. Twenty-nine age-matched subjects were used as a control. Blood pressure was measured in the sitting position using an upright standard sphygmomanometer. Blood was drawn from arteriovenous shunt just before starting HD or from the antecubital veins of controls for determination of blood chemistry as described previously.^14^ GDF11 was measured by a commercially available enzyme-linked immunosorbent assay (ELISA) system (Wuhan EIAab Science Co. Ltd.). Informed consent was obtained from all subjects, and the study protocol was approved by the Institutional Ethics Committees of Kurume University School of Medicine, Japan. This work was conducted in accordance with the Declaration of Helsinki.

Data were presented as mean values ± standard deviation or medians with the interquartile range. The medication for hypertension and dyslipidemia (renin–angiotensin system [RAS] inhibitors and statins) and the presence or absence of diabetes mellitus were coded as dummy variables. Statistical differences in clinical parameters were determined using Student's *t*-test or chi-squared test. Correlations between GDF11 and other clinical variables were determined by a linear regression analysis. To determine the independent determinants of GDF11, multiple stepwise linear regression analysis was performed. Statistical significance was defined as *p* < 0.05. All statistical analyses were performed with the use of the SPSS system (SPSS, Inc.).

## Results

Table 1 shows the clinical variables of subjects. Serum GDF11 levels in HD patients were significantly higher than those in controls (9.4 ± 5.1 pg/mL vs. 7.3 ± 5.9 pg/mL). Hemoglobin, albumin, and low-density lipoprotein cholesterol levels were significantly lower in HD patients, whereas triglycerides, blood urea nitrogen creatinine, uric acid, corrected calcium, phosphate, intact parathyroid hormone, and high-sensitivity C-reactive protein values were higher than in control subjects. The number of HD patients with diabetes or who received RAS inhibitors or statins were 10, 47, and 2, respectively. A statistical significance was demonstrated between GDF11 and hemoglobin (inversely) (Table 2). Multiple stepwise regression analysis revealed that hemoglobin (*p* < 0.001) was a sole independent correlate of GDF11 levels in HD patients (*R*^2^ = 0.168) (Table 2).

**Table 1.** **Clinical Characteristics of Subjects**

	Controls	HD patients	*p*
No. of patients	29	62	
No. of men/women	4/25	34/28	<0.001
Age (years old)	56.2 ± 3.9	52.6 ± 13.7	0.062
HD duration (months) (range)		7.7 ± 5.2 (0.5–23)	
Body mass index (kg/m^2^)	21.8 ± 2.4	21.5 ± 4.7	0.732
Systolic BP (mmHg)	131.6 ± 28.50	140.4 ± 22.0	0.114
Diastolic BP (mmHg)	75.3 ± 20.2	82.6 ± 12.6	0.148
Hemoglobin (g/dL)	13.6 ± 2.0	11.2 ± 1.3	<0.001
Albumin (g/dL)	4.5 ± 0.2	3.6 ± 0.5	<0.001
LDL cholesterol (mg/dL)	120.6 ± 32.2	87.2 ± 30.9	<0.001
Triglycerides (mg/dL)	82.3 ± 72.5	128.69 ± 95.6	0.024
BUN (mg/dL)	14.0 ± 3.2	59.5 ± 12.0	<0.001
Serum Cr (mg/dL)	0.6 ± 0.1	11.4 ± 2.2	<0.001
Uric acid (mg/dL)	4.5 ± 1.1	7.4 ± 1.0	<0.001
Corrected Ca (mg/dL)	8.8 ± 0.2	9.6 ± 0.9	<0.001
Phosphate (mg/dL)	3.6 ± 0.5	5.4 ± 1.1	<0.001
Intact PTH (pg/mL)	50.2 ± 12.9	155.2 ± 97.0	<0.001
No. of DM (±)	29/0	52/10	0.024
HbA1c (%)	5.2 ± 0.5	5.1 ± 0.8	0.197
hsCRP (mg/dL)	544 ± 785	3704 ± 11681	0.040
GDF11 (pg/mL)	7.3 ± 5.9	9.4 ± 5.1	0.043
Medication			
RAS inhibitors (±)	29/0	15/47	<0.001
Statins (±)	29/0	60/2	0.328

Values are shown as mean ± standard deviation or range.

BP, blood pressure; BUN, blood urea nitrogen; Ca, calcium; Cr, creatinine; DM, diabetes mellitus; GDF11, growth differentiation factor 11; HbA1c, glycated hemoglobin; HD, hemodialysis; hsCRP, high-sensitivity C reactive protein; LDL, low-density lipoprotein; PTH, parathyroid hormone; RAS, renin–angiotensin system.

**Table 2.** **Univariate and Multiple Stepwise Regression Analysis for the Correlates of GDF11 Levels in HD Patients**

	Univariate			Multiple stepwise regression		
Variables	*β*	SE	*p*	*β*	SE	*p*
Age	−0.177	0.047	0.168			
Sex	−0.043	1.315	0.738			
HD duration	−0.151	0.129	0.249			
Systolic BP	−0.104	0.030	0.422			
Body mass index	−0.131	0.140	0.312			
Hemoglobin	−0.447	0.457	0.001	−0.447	0.457	0.001
Albumin	−0.041	1.451	0.754			
Triglycerides	−0.138	0.007	0.284			
LDL cholesterol	−0.248	0.021	0.054			
BUN	0.033	0.055	0.800			
Serum Cr	−0.012	0.340	0.926			
Uric acid	−0.106	0.676	0.412			
Corrected Ca	−0.090	0.741	0.487			
Phosphate	−0.037	0.607	0.778			
Intact PTH	0.052	0.007	0.691			
HbA1c	−0.091	0.801	0.491			
DM	−0.115	1.770	0.372			
hsCRP	0.112	0.000	0.396			
RAS inhibitors	−0.202	1.246	0.407			
Statins	0.580	1.550	0.662			

*β*, standardized regression coefficients. *R*^2^ = 0.168.

Male = 0, female = 1. Medication (+) = 0, medication (−) = 1.

SE, standard error.

## Discussion

Cardiac hypertrophy and remodeling as well as impaired regeneration of skeletal muscle and neurons in old mice have been reversed by heterochronic parabiosis.^4–7^ Furthermore, circulating levels of GDF11 are decreased with age in several animal species, and supplementation of GDF11 has been shown to rescue these age-related organ damage.^4–7^ These observations suggest that GDF11 may be a circulating rejuvenation factor in young mice that could reverse age-related dysfunction of multiple organ systems.^4–7^ However, other groups have reported that GDF11 is increased *rather than* decreased with age and that reduced circulating levels of GDF11 are unlikely a rejuvenation factor that reverses age-dependent changes in mouse heart, skeletal muscle, and brain.^9–11^ Therefore, to further examine the pathophysiological role of GDF11 in human aging process, we chose uremic patients and measured their GDF11 levels in this study because age-related organ damage is accelerated under uremic conditions.^12,13^

We demonstrated here for the first time that circulating GDF11 levels were significantly increased in HD patients than in age-matched controls and that age was not associated with GDF11 levels in uremic subjects. Recently, Fadini et al. reported that compared with control subjects, diabetic patients had significantly increased plasma GDF11 values, especially those with macroangiopathy.^15^ Moreover, they showed that there was no correlation between age and GDF11 in type 2 diabetic subjects.^15^ These findings were in striking contrast with the observation in the Heart and Soul and HUNT3 cohorts, showing that GDF11/8 values were lower in older participants and inversely associated with increased risk of cardiovascular events and overall morality in patients with IHD.^8^ As the case with uremia, given that diabetes is characterized with accelerated aging and increased risk for cardiovascular disease,^16,17^ kinetics and regulation of circulating GDF11 may differ between normal physiological aging process and accelerated pathological aging conditions, such as uremia and diabetes. Elevation of GDF11 levels in uremic patients could be attributed to impaired clearance by HD, which may work as a counter-system against accelerated aging-related organ damage in patients with HD. Another possibility is that difference of reagents and/or methods used for assaying GDF11 could account for the discrepant results (modified aptamer-based proteomic platform in the Heart and Soul and HUNT3 cohorts vs. ELISA in Fadini's and our subjects).

In this study, we also found that low hemoglobin values were independently associated with high GDF11 levels in HD patients. GDF11 has been shown to inhibit erythroid maturation in mice both *in vivo* and *ex vivo*.^18,19^ So increase of GDF11 levels may be involved in erythropoietin-resistant anemia, which is often observed in uremic patients.^20^ Since a poor initial hematopoietic response to erythropoietin is associated with adverse cardiovascular events in diabetic patients with uremia,^20^ pharmacological upregulation and/or supplementation of GDF11 may not exert beneficial effects on aging-related disorders, such as cardiovascular disease in HD patients.

## Limitations

There are some limitations in this study. This study was cross-sectional and thus could not assess the questions of whether elevation of GDF11 was a cause or consequence of uremia or anemia. The small sample size is limited for subgroups analysis with solid results. Hemoglobin levels in HD patients are influenced by therapies, such as administration of erythropoiesis-stimulating agents and iron. Therefore, various comedications may limit and confound the present findings. Thus, a further study will be needed to address whether supplementation of GDF11 could be functionally correlated with uremia-related organ dysfunction.

## Abbreviations Used

ELISA: enzyme-linked immunosorbent assay
GDF11: growth differentiation factor 11
HD: hemodialysis
IHD: ischemic heart disease
RAS: renin–angiotensin system
